# Memories Are Made of This: Modeling the CaMKII Molecular Switch

**DOI:** 10.1371/journal.pbio.0030124

**Published:** 2005-03-29

We all know that our memories are stored somehow in our brains. But exactly how do we remember the way to our office or what our mother looks like or the date we got married? Scientists attribute our ability to store apparently infinite numbers of memories for decades to long-lasting changes in the electrical, structural, and biochemical properties of neurons. One cellular mechanism proposed to be involved in the storage of memories—long-term potentiation—involves alterations in the strength of messages passed from one neuron to another across structures known as synapses.

The initiation of long-term potentiation is caused by activation of n-methyl-d-aspartate receptors on the receiving neuron and a subsequent increase in the intracellular calcium concentration in a region of the neuron that is called the postsynaptic density. The increase in calcium, in turn, activates the calcium/calmodulin-dependent protein kinase II (CaMKII). This enzyme seems to play a critical role in long-term potentiation, and has been proposed as one of the leading candidates to act as the molecular switch that maintains stable synapse-specific cellular changes. To fulfill this role, CaMKII would need to have stable UP and DOWN positions, or states, much like a light switch.

Xiao-Jing Wang and colleagues now provide a new analysis that strengthens the argument that CaMKII is a molecular switch involved in the storage of long-term neural changes. The activity of the CaMKII holoenzyme (the complete enzyme consisting of both regulatory and catalytic subunits) is controlled by its autophosphorylation state—the enzyme is able to add phosphate groups to specific amino acids within itself. Previous modeling studies have shown that the interplay between the autocatalytic addition of phosphate groups to CaMKII and the removal of phosphate groups by protein phosphatase-1 (PP1) enzymes produces two stable states of the CaMKII enzyme at basal free calcium levels. The DOWN state is unphosphorylated; the UP state is highly phosphorylated. When there is a transient high input of calcium, as happens when long-term potentiation is induced, the CaMKII enzyme flips from a DOWN state to a persistent UP state.

The questions that Wang and colleagues have now asked are what factors affect the stability of the state of this switch, and how many CaMKII holoenzymes are needed to construct a switch that could last a lifetime. These questions are important because a switch that could be spontaneously reset by small, random fluctuations of the conditions within the postsynaptic density would not be useful in maintaining stable long-term changes. The researchers have used a mathematical probabilistic modeling technique known as Monte Carlo simulation, together with the known biochemical and thermodynamic characteristics of CaMKII and PP1, to test how random fluctuations in the chemical reactions involved in the CaMKII/PP1 system change the state of the switch.

They report that switch state stability requires a balance between the phosphorylation and dephosphorylation rates of CaMKII, and that the turnover rate of the kinase—the replacement of old molecules with new ones—critically affects switch stability. However, their main finding is that the lifetime of states of the switch increases exponentially with the number of CaMKII holoenzymes that are present. This finding is important because experimental work by other researchers has estimated that there are about 30 CaMKII holoenzymes present in a typical postsynaptic density, and until Wang's team did their modeling it was unclear whether this number of holoenzymes could build a switch stable enough to last a lifetime. In fact, Wang and co-workers estimate that a switch containing as few as 15 holoenzymes can remain activated for longer than a human lifetime. Thus, the researchers conclude, CaMKII switches may indeed play a critical role in preserving our precious memories throughout our lives.

